# Evidence of concurrent and predictive criterion validity of the Child Communication Screening Instrument from 0 to 36 months (IRC-36)

**DOI:** 10.1590/2317-1782/20232022218en

**Published:** 2023-12-22

**Authors:** Dayanne Priscila Rodrigues de Almeida, Ana Augusta de Andrade Cordeiro, Larissa Nadjara Alves Almeida, Camila Arruda Manchester de Queiroga, Bianca Arruda Manchester de Queiroga

**Affiliations:** 1 Programa de Pós-Graduação em Saúde da Comunicação Humana, Universidade Federal de Pernambuco – UFPE - Recife (PE), Brasil.; 2 Departamento de Fonoaudiologia, Universidade Federal de Pernambuco – UFPE - Recife (PE), Brasil.; 3 Curso de Fonoaudiologia, Centro Universitário de João Pessoa – UNIPÊ - João Pessoa (PB), Brasil.

**Keywords:** Child Language, Child Development, Mass Screening, Validation Study, Psychometrics, Speech, Language and Hearing Sciences

## Abstract

**Purpose:**

To determine evidence of concurrent and predictive criterion validity of the Communication Screening Instrument for children aged 0 to 36 months (IRC-36).

**Methods:**

78 parents/guardians of children who attend the childcare service of the Family Health Centers participated in the research, in addition to 33 children aged between 0 and 36 months, invited to the second stage of the study. In its first stage, 13 health professionals were trained to apply the IRC-36 to the children's parents/guardians. In the second moment, the parents responded to a new IRC-36 application, and the children were evaluated with Denver II.

**Results:**

IRC-36 correlated with Denver II in more than half of the cases, confirming the instrument’s concurrent criterion validity. IRC-36 results in the first stage did not significantly correlate with Denver II. The instrument's cutoff value was 12, which is the reference value between children at risk and not at risk of communication disorders. The instrument had high sensitivity and an accuracy value within the recommended levels. The occurrence of risk of communication changes was higher in the second IRC-36 application.

**Conclusion:**

The study presented evidence of concurrent criterion validity, indicating that the instrument has evidence of accuracy and validity measures to screen communication in children aged 0 to 36 months, being able to identify the risk for communication disorders.

## INTRODUCTION

Following up on children’s growth and development is one of the main approaches indicated by the Ministry of Health in their pediatric healthcare strategy^(1)^. In the context of development surveillance, hearing and language development are important milestones to be monitored in child development follow-up, along with motor development^(2)^.

Screening instruments are important tools to identify developmental delays. However, due to the lack of precise and reliable instruments, they are normally not used to monitor child language development^(3)^. There are some child language assessment instruments in Brazil, but screening ones are still scarce^(4)^.

Screening tests identify people with an impairment or disease who are not presenting symptoms yet, thus making diagnosis and treatment easier and minimizing the long-term effects of the condition^(5)^. In the case of children, the absence of early identification and the consequent late diagnosis hinder their access to treatment and cause greater impairment in their quality of life and later development^(6)^.

The great advantages of screening tests are that they are quick and easy to apply and are usually inexpensive, posing little risk to the individual and ensuring results with good sensitivity^(7)^. These tests must comprise health professionals’ clinical practice^(8)^. However, they must be developed considering psychometric measures, such as validity and accuracy^(9,10)^.

The Child Communication Screening Instrument from 0 to 36 months (IRC-36) was developed to screen child communication in the first 3 years of life. It is a simple, parent-report instrument, divided into nine categories, distributed and organized per age range, with specific questions for each phase of the children’s development from 0 to 36 months. After applying it, the professional can identify the child’s communication in that stage, classifying their performance as at risk, under observation, or as expected^(11)^.

An instrument must have its psychometric properties verified for it to be recommended^(12)^. IRC-36 underwent the development and content validation stages^(11)^. This study aims to verify other validity aspects. In the initial stages^(11)^, IRC-36 had a high interrater agreement index, confirming its content validity.

Validity is one of the important psychometric measures and is divided into three types: content validity, criterion validity, and construct validity^(8)^. Criterion validity is verified when the result of the test being validated can be compared with another gold-standard test^(8)^; it is divided into two subtypes: predictive and concurrent^(10)^. Predictive criterion validity is related to the test result concerning the future condition of the person being assessed – i.e., whether the instrument can predict future performance. In concurrent criterion validity, the results of the test being validated are compared with the results of another test applied along with it^(10)^.

Accuracy is another important psychometric measure. It is related to the sensitivity and specificity values of the test being validated in comparison with a reference test^(10)^.

Given the importance of psychometric measures to validate an instrument^(9)^ and its validation stages^(10)^, this study aimed to continue the validation of IRC-36, determining evidence of its criterion validity and accuracy (sensitivity and specificity).

## METHODS

This research was approved by the Ethics Committee of the Department of Health Sciences of Federal of Pernambuco University under number CAAE: 26176319.6.0000.5208, complying with the resolution of the National Health Council. All participating individuals were informed of the research and signed an informed consent form. This is a quantitative, exploratory, instrument validation study.

The study was conducted in two stages. The first one was carried out in four Family Health Centers, selected by convenience. Their participation was authorized by the Municipal Department of Health. First, the research was presented in the family health team’s meeting, and training was provided to the professionals who wished to participate in it – 10 community health agents (CHA) and three nurses. The training was scheduled at the unit where the professionals worked, and they had to attend it to learn how to apply IRC-36. It consisted of reading and explaining each IRC-36 item, when CHAs and nurses also had their questions answered to ensure they would be confident to apply it.

After the training, the professionals applied IRC-36 to the children’s parents/guardians in their work routine – i.e., nurses in neonatal care and CHAs in home visits.

IRC-36 is divided into nine categories, each one comprising an age range, as follows: **category 1**: 0 to 3 months; **category 2**: 4 to 6 months; **category 3**: 7 to 9 months; **category 4**: 10 to 12 months; **category 5**: 13 to 15 months; **category 6**: 16 to 18 months; **category 7**: 19 to 24 months; **category 8**: 25 to 30 months; **category 9**: 31 to 36 months).

The categories have 10 closed-ended questions, to which they can be answered with either yes, no, or sometimes. “Yes” answers score 2 points, “sometimes” answers score 1 point, and “no” answers do not score. At the end of each question, room is provided for complementary answers, whenever it is answered “yes “ or “sometimes”. The total score for each category is 20 points, which is the best result. The final results are as follows: **“at risk”**, for children who scored 10 or less; **“under observation”**, for those who scored 11 to 14 points, and **“out of risk”** for those who scored 15 to 20 points.

When they had finished applying them, the instruments were sent to the researching speech-language-hearing (SLH) therapist for later analysis. Altogether, 92 assessments were sent, although 23 had to be excluded because they were not correctly filled out. Hence, 69 instruments remained in the first stage.

The second stage of the study aimed to verify the concurrent and predictive criterion validity. Thus, the parents/guardians were contacted to reapply IRC-36, and their children were submitted to the Denver II Developmental Screening Test.

Only 24 of all parents/guardians who participated in the first stage returned for the second one. There were various reasons for the sample loss, especially that the patient had moved to another region, they could not be reached, or they did not attend the scheduled assessment.

The assessments were conducted on the same day, one after the other, at the Family Health Centers or the child’s home, after the parents/guardians had signed the informed consent form. The researching SLH therapist was blind in the second stage assessment concerning the results obtained in the first IRC-36 application and Denver II test.

### Denver II

Denver II assesses children 0 to 6 years old regarding their development in the following domains: personal social (child’s socialization), fine motor (eye-hand coordination), language (recognizing, understanding, and using language), and gross motor (body motor control), verified by directly observing the child.

Denver II items must be visualized while the child performs the tasks, with the following possible results: pass (P), fail (F), or refused (R). If they are not observed doing the task, some items allow the parent/guardian to report whether the child already performs it (if they had the opportunity to perform it). According to Denver II, children can have normal development or be at risk of delay.

The SLH therapist had to be trained to apply Denver II, in a 70-hour course that certified her to apply the instrument. She received theoretical and practical training in online classes, with article reading and discussion, explanatory videos, and practical exercises. She also had to acquire the standardized “test kit” to avoid decreasing result reliability.

After applying the tests, the parents received the results and were instructed to stimulate oral language. When necessary, the child was referred to specialized healthcare.

This study was conducted in a municipality in the Metropolitan Region of Recife. Three nurses and 10 CHAs applied the instrument in the first stage, and the researching SLH therapist applied it in the second stage. Altogether, the study had 78 participating parents/guardians – mostly mothers (99%) – of children who attended the service where these professionals worked. Also, 33 children aged 0 to 36 months were invited to the second stage of the study.

The 78 IRC-36 protocols that were applied referred to 41 (52.6%) female children and 37 (47.4%) male children. The mean time between the first and second IRC-36 applications was 5 months and 3 days. The applications are described in Chart 1.

**Chart 1:** Application of the instruments per professional, place of application, and number of instruments applied

Examiner	Place of application	IRC-36 application (1^st^ stage)	IRC-36 application + Denver II (2^nd^ stage)		Total
			IRC-36 reapplication + Denver II	IRC-36 application + Denver II	
CHA	Home (home visit)	57	-	-	57
Nurse	Family Health Center (neonatal care)	12	-	-	12
SLH therapist	Family Health Center or home	-	24	9	33
Total		69	24	9	102

**Caption**: CHA = community health agent; SLH = speech-language-hearing; IRC-36 = Child Communication Screening Instrument from 0 to 36 months

The study exclusion criteria were parents/guardians with intellectual disability, parents/guardians under 18 years old, parents/guardians of children with genetic or neurological changes, and children diagnosed with hearing and/or visual impairments.

Collected data were organized and analyzed in SPSS statistical program, version 21.0. After verifying the non normal data distribution, the Fisher Exact and Spearman Correlation tests were applied to assess associations and correlations. The level of significance for calculations was set at 5%.

Only 24 out of the 69 parents who answered the first IRC-36 attended its reapplication and Denver II application. Also, another nine children joined the sample whose parents answered IRC-36 only in the second application, along with Denver II. Hence, there was a total of 102 IRC-36 and 33 Denver II tests applied.

**Figure 1:**
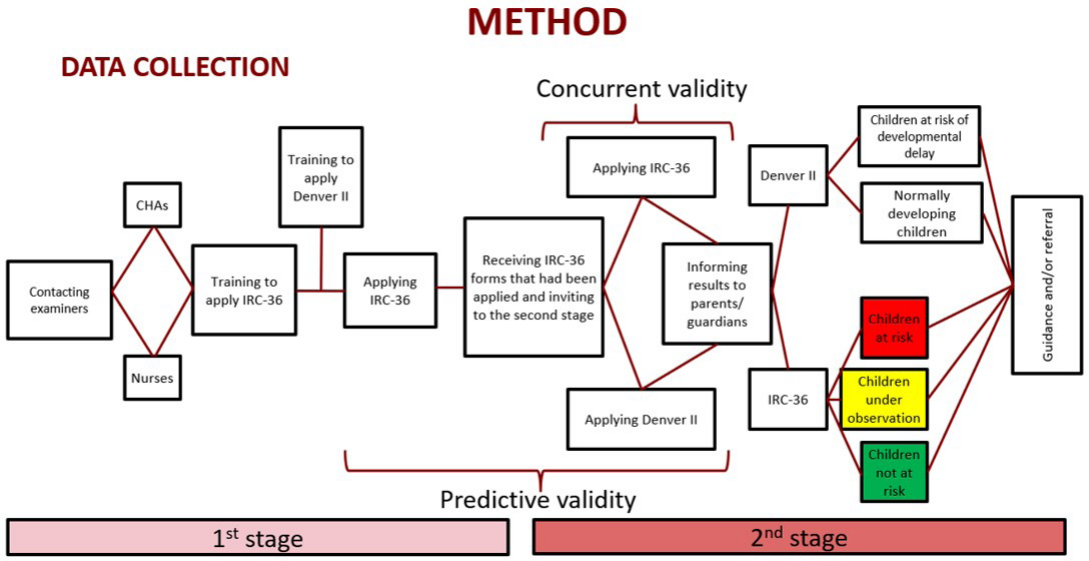
Methodological data collection flowchart

Source: The author (2022)

It is important to highlight that, given the COVID-19 pandemic experienced in the last years, the beginning of the collection was delayed, and its pace was slowed by the difficulties faced during the pandemic. Nevertheless, despite all difficulties, enough data were collected to finish the research. The data collection flowchart is shown below in Figure 1.

## RESULTS

Table 1, below, crosses the children’s performance in IRC-36 1^st^ (first application, conducted by the CHAs and nurses), IRC-36 2^nd^ (second application, conducted by the lead researcher), and Denver II. The analysis considered “expected” the performance of children **not at risk** and “not expected” the performance of children whose IRC-36 scores indicated they were **under observation** or **at risk** of communication changes.

**Table 1:** Data on the children’s performance classification in IRC-36 and total Denver II

**Variable**		**DENVER II RESULT**						**p-value**
		**NORMAL**		**RISK**		**Total**		
		**n**	**%**	**N**	**%**	**N**	**%**	
IRC-36 FINAL 1	Not at risk	7	58.3%	7	58.3%	14	58.3%	0.435
	Under observation	4	33.3%	2	16.7%	6	25.0%	
	At risk	1	8.3%	3	25.0%	4	16.7%	
	Total	12	50.0%	12	50.0%	24	100.0%	
IRC-36 FINAL 2	Not at risk	**15**	**83.3%**	4	26.7%	19	57.6%	0.002
	Under observation	3	16.7%	**6**	**40.0%**	9	27.3%	
	At risk	0	0.0%	5	33.3%	5	15.2%	
	Total	18	54.5%	15	45.5%	33	100.0%	
IRC-36 1^st^	Expected for the age	11	91.7%	9	75%	20	83.3%	0.295
	Not expected	1	8.3%	3	25%	4	16.7%	
	Total	12	100.0%	12	100.0%	24	100.0%	
IRC-36 2^nd^	Expected for the age	**15**	**83.3%**	4	26.7%	19	57.6%	0.0001
	Not expected	3	16.7%	**11**	**73.3%**	14	42.4	
	Total	18	54.5%	15	45.5%	33	100.0%	

Fisher Exact test; significance at p < 0.05*

**Caption:** IRC-36 FINAL 1 = result from the three IRC-36 stratifications in the first application; IRC-36 FINAL 2 = result from the three IRC-36 stratification in the second application; IRC-36 1^st^ = IRC-36 in the first application; IRC-36 2^nd^ = IRC-36 in the second application

The findings show that the children’s performance in the second IRC-36 application was associated with Denver II’s classification in 83.3%. This means that those classified as not at risk by IRC-36 were also within the normal range in Denver II – thus confirming the instrument’s concurrent validity.

Moreover, the frequency of cases whose IRC-36 performance was “not expected” for their age (including at-risk and under observation) was significantly associated (73.3%) with the cases at-risk in Denver II. These results show that IRC-36 has good sensitivity and reinforce its concurrent criterion validity.

On the other hand, as seen in Table 1, no significant associations were found between the scores obtained in the first IRC-36 application and Denver II.

A Spearman Correlation analysis (shown in Table 2, below) was performed to verify more in-depth whether different results would be obtained by maintaining the original IRC-36 scores, not considering the classification of children **under observation** and **at risk** as “not expected”.

**Table 2:** Correlation matrix (Spearman's rho) between the performance in the first and second IRC-36 applications and Denver II and the language domain in Denver II

Tests	Denver II	Denver II – Language
IRC-36 1^st^	0.752	0.715
IRC-36 2^nd^	0.606**	0.621**

** The correlation is significant at p < 0.01

Once again, a strong correlation was found between the second IRC-36 application and Denver II (rho = 0.606), confirming the concurrent validity that the instrument assesses aspects of language and can be compared with Denver II, which is a reference measure. The applications also had a strong correlation with the specific language areas in Denver II (rho = 0.621; p < 0.01). As in the previous situation, again the first IRC-36 application was not significantly correlated with Denver II.

There was no significant correlation between IRC-36 1^st^ results and Denver II in the standard classification and the “expected” and “not expected” results. Therefore, these results do not confirm the predictive capacity of the instrument.

Denver II has the following domains: personal-social, fine motor, language, and gross motor. Hence, another association analysis was performed with the Fisher Exact test, as seen in Table 3, to further explore the relationships between IRC-36 and the language domain in Denver II.

**Table 3:** Data on the children’s performance classification in IRC-36 and the language domain in Denver II

**Variable**		**RESULT DENVER II LANGUAGE**						p-value
		**NORMAL**		**RISK**		**Total**		
		**N**	**%**	**N**	**%**	**N**	**%**	
IRC-36 FINAL 1	Not at risk	**11**	**61.1%**	3	50.0%	14	58.3%	0.853
	Under observation	4	22.2%	2	33.3%	6	25.0%	
	At risk	3	16.7%	1	16.7%	4	16.7%	
	Total	18	75.0%	6	25.0%	24	100.0%	
IRC-36 FINAL 2	Not at risk	**15**	**83.3%**	4	26.7%	19	57.6%	0.002
	Under observation	3	16.7%	**6**	**40.0%**	9	27.3%	
	At risk	0	0.0%	5	33.3%	5	15.2%	
	Total	18	54.5%	15	45.5%	33	100.0%	
IRC-36 1^st^	Expected for the age	15	83.3%	5	83.3%	20	63.6%	0.749
	Not expected	3	16.7%	1	16.7%	4	36.4%	
	Total	18	75%	6	25%	24	100.0%	
IRC-36 2^nd^	Expected for the age	**18**	**94.7%**	1	5.3%	19	57.6%	0.002
	Not expected	6	42.9%	**8**	**57.1%**	14	42.4%	
	Total	24	72.7%	9	27.3%	33	100.0%	

Fisher Exact test; significance at p < 0.05*

**Caption:** IRC-36 FINAL 1 = result from the three IRC-36 stratifications in the first application; IRC-36 FINAL 2 = result from the three IRC-36 stratification in the second application; IRC-36 1^st^ = IRC-36 in the first application; IRC-36 2^nd^ = IRC-36 in the second application

The relationship between IRC-36 classification and the Denver II language domain showed a similar result, with an even greater association (94.7%) when cases whose development was “expected” and “not expected” were observed regarding normality and risk. As previously observed, again the first IRC-36 application was not significantly correlated with Denver II in the language domain.

The study also verified whether the instrument’s cutoff scores to discriminate individuals with the proposed outcomes would be confirmed. It considered “expected” those whose results were **not at risk** and “not expected” when they were **under observation** or **at risk**.

**Figure 2:**
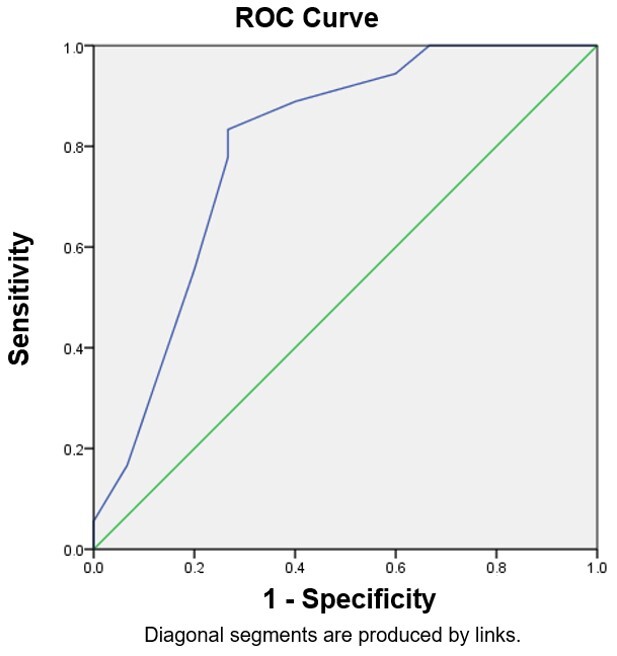
ROC curve in the second IRC-36 application, based on the Denver II classification – normal and at-risk

Source: The author (2022)

Figure 2 shows the ROC curve with an area under the curve of 0.796 (95% CI: 0.634 – 0.959).

The best cutoff value was set at the point with the least possible error and the best sensitivity measure, which was 11.5. However, since IRC-36 scores use whole numbers, the cutoff was set at 12. Considering that IRC-36 is a screening instrument, it will indicate the risk of communication changes when its score is below 12 and the absence of risk of such changes when it is equal to or above 12.

The test accuracy is represented by the area of the curve, which was 79.6% (Table 4) – i.e., within recommended levels, thus confirming that the instrument can predict whether a child is at risk of communication changes. The instrument sensitivity was 94.4%, which is high for screening instruments, as it verifies its capacity to detect people who probably have the investigated condition.

**Table 4:** Area under the curve and coordinates of the IRC-36 2^nd^ ROC curve

**Area**	**Std. Error^a^**	**Significance level P (Area=0.5)^b^**	**Asymptotic 95% confidence interval**		**Associated criterion**	**Sensitivity (%)**	**Specificity (%)**
			**Lower limit**	**Upper limit**			
0.796	0.083	<0.004	0.634	0.959	11.50	94.4	60.0

a DeLong et al.^(13)^

b Binomial exact

In the first IRC-36 application, many children (13%) were classified as “under observation” and “at risk” of communication changes, which increased to 15.2% in the second application. Considering the result as **not expected** – which sums both “at risk” and “under observation” –, these values increased respectively to 36.2% and 42.5%.

Concerning the Denver II domains, the language had the most children at risk of delay (27.3%), followed by personal social (24.2%). Considering the overall Denver II result – i.e., all instrument domains together –, the number of children at risk of developmental changes was 45.5%.

**Figure 3:**
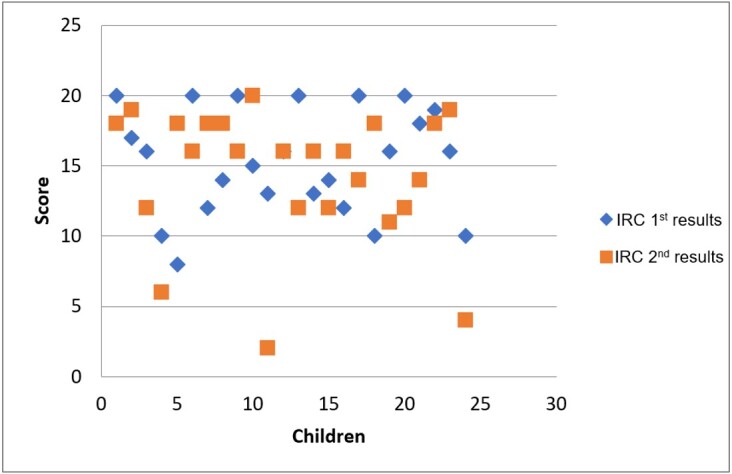
Performance of the 24 children in the first and second IRC-36 applications over time

Source: The author (2022)

The performance over time of the 24 children who were addressed in the two IRC-36 applications is shown in Figure 3.

Encompassing the children assessed in the different moments, four out of the 24 were “at risk” and six “under observation” in the first IRC-36 application, while in the second one three were “at risk” and seven “under observation”. Two children who were “at risk” in the first IRC-36 application remained so in the second one, one who was “under observation” decreased to “at risk”, and one remained “under observation”. The means in the two moments were 14.95 and 14.37 – i.e., lower in the second IRC-36 application.

Two out of the 33 children screened with IRC-36 and Denver II were referred for specialized healthcare because they were “at risk” in both assessments and had considerable language delay and signs of autism spectrum disorder, such as not responding when called by name, interaction difficulties, vocal stereotypies, and lack of eye contact.

## DISCUSSION

The results of this research indicate a positive correlation between IRC-36 and Denver II, confirming the concurrent criterion validity. Denver II is an internationally recognized instrument that is widely used in clinical practice and research in Brazil^(14,15)^ and has been undergoing validation stages with positive results^(16,17)^. Hence, its correlation with IRC-36 shows how valid the new instrument is and how useful it can be to verify communication aspects, given the scarcity of child language screening instruments in Brazil^(4)^.

The verification of predictive criterion validity did not obtain significant values – i.e., in this investigation, the instrument could not predict the children’s future communication performance. Nevertheless, some aspects may have interfered with this result, particularly the time between the first and second applications, the different examiners who applied the instrument in the two stages, and conducting the research during a critical moment of the COIVID-19 pandemic – which may have led to changes in the children’s development. Studies have already demonstrated that social isolation during the COVID-19 pandemic may cause risk factors for child growth and development^(18)^.

Another issue regarding the application that must also be considered is that the training provided to the examiners may not have been enough to train them thoroughly, thus compromising the final results when both applications were compared. This may have been due to its methodology, which consisted only of lectures, whereas the recommended for continuing education is to give priority to active methodologies, in which professionals experience practical aspects, making it easier for them to acquire knowledge^(19)^. However, it was not possible to train them in another format because of the routine at the health service, the pandemic during which data were collected, and the little time (which always poses a challenge in primary healthcare)^(20)^.

The IRC-36 sensitivity value in the second moment when related to Denver II in the language domain showed that IRC-96 correctly identified 94.4% of the cases at risk of changes confirmed with Denver II. A Dutch study verified the concurrent and predictive validity of a child language screening test and found good concurrent validity results (0.79 sensitivity and 0.86 specificity) and high predictive capacity (0.82 sensitivity and 0.74 specificity). This indicates how important these instruments can be to observing child language, predicting future results, and providing support to pediatric health professionals^(21)^. Hence, IRC-36 can be used to furnish information on children’s communication status and help professionals follow up on child development.

The ROC curve demonstrated the cutoff score among children at risk and not at risk of changes, with a 79.6% accuracy value, which is within indicated levels. The farther the curve is from the main diagonal, nearing the left upper corner, the better the test’s performance to discriminate people with and without the investigated outcome^(22)^.

The instrument’s cutoff was verified with the ROC curve, identifying that the score that distinguishes individuals at risk and not at risk of communication changes is 12. This refers to the primary IRC-36 stratification value^(11)^, which defines children “at risk” of communication changes as those who score up to 10 and “under observation” as those who score from 11 to 14. However, given the ROC curve results that delimited the value between children at risk and not at risk, those “under observation” were stratified at values from 13 to 14, indicating that their communication development must be monitored. Cutoff scores may be better verified with larger samples, following the original classification.

Since the main construct of IRC-36 is communication, its application may be briefer than that of Denver II, which encompasses four development domains^(23)^.

Moreover, IRC-36 is a low-cost screening test, requiring only the form to be filled out and previous instructions^(11)^, meeting the recommendations for screening instruments (quick, easy to apply, and low-cost)^(7)^. In this regard, it is different from Denver II, which requires specific training for its application and the acquisition of the test kit, with specific assessment items^(23)^.

The instrument being validated can be applied by health and education professionals involved with child development follow-up to help them verify the children’s communication development. It is important to highlight that language delays may be highly prevalent in the first 3 years of life^(24)^.

The risk of communication changes occurred in 13% and 15.2% of the subjects respectively in the two moments. These results demonstrate that child communication aspects must be followed up, as current changes may have great future consequences. Also, failing to identify language changes at the right time may influence child development in all areas^(25)^.

The number of children at risk of changes was high in both IRC-36 and Denver II. A study in preschoolers assessed with Denver II verified that 24.8% of them were at risk of developmental delay^(26)^, which was almost twice as much in the present research (45.5%). This corroborates a study in primary healthcare users aged 7 to 18 months^(27)^, in which 47.37% of children were at risk. This high percentage at risk of developmental delay may be related to the reality of the children followed up in primary healthcare in the context approached in the present investigation. They may receive little stimulation due to many family circumstances, and such stimulation opportunities may even decrease as they grow older^(27)^. Furthermore, as previously mentioned, the research was carried out during a critical pandemic period, in which children had limited social experiences due to social isolation, which may have increased the prevalence of children at risk^(18)^.

Other studies also verified language as the area at the greatest risk of developmental delay^(27,28)^. Thus, it is important to monitor linguistic development and instruct caregivers, considering that language acquisition is related to the stimuli the children receive in the environment to which they belong^(29)^.

IRC-36 can be useful to implement strategies to stimulate child communication when it is “under observation” or “at risk”. Intervention strategies must be provided to the children and their families to prevent developmental delay, as inadequate family stimulation is a risk factor for language delay^(30)^.

IRC-36 is a useful instrument that has passed the indicated validation stages, obtaining positive values that demonstrate the instrument's validity. This study verified other validity parameters that the instrument passed with satisfactory results.

The instrument can help verify child communication in primary healthcare and possibly in other contexts, such as daycare centers. It is a simple, easy-to-apply test, with important applicability to child development, as language is a skill that influences other areas of development^(31)^.

## CONCLUSION

The study showed a strong correlation between IRC-36 and Denver II, confirming its concurrent criterion validity and indicating that the instrument being validated can be used to screen communication in children aged 0 to 36 months. The instrument also obtained satisfactory accuracy and sensitivity measures, indicating that it can identify the risk for communication changes. It verified a striking number of children “under observation” and “at risk” of communication changes, indicating how important it is to follow up on child development. Predictive criterion validity values were not significant. However, some methodological and circumstantial aspects may have influenced the results, which must be better investigated in future studies.

Considering the COVID-19 pandemic when the research was conducted, the difference in time between the first and second applications, and that it was applied by different professionals, it is suggested that future studies apply the instrument after the pandemic to confirm the previous results. Moreover, they should decrease and standardize the time between applications in the two stages, with larger samples, also diversifying the social and geographical context of the study population. The present research was conducted only in primary healthcare with families of a low socioeconomic level. Another relevant suggestion is to verify which difficulties examiners had and the mean time they took applying the screening instrument.
