# Supplementary material for: Evidence of concurrent and predictive criterion validity of the Child Communication Screening Instrument from 0 to 36 months (IRC-36)
Source: Codas. 2023 Dec 22;36(1):e20220218. doi: 10.1590/2317-1782/20232022218en (PMC10750861; doi:10.1590/2317-1782/20232022218en)
Supplement: Translation

# Translation: Evidências de validade de critério concorrente e preditiva do Instrumento de Rastreio da Comunicação de Crianças de 0 a 36 meses (IRC-36)

**DOI:** 10.1590/2317-1782/20232022218pt

**Authors:** Dayanne Priscila Rodrigues de Almeida, Ana Augusta de Andrade Cordeiro, Larissa Nadjara Alves Almeida, Camila Arruda Manchester de Queiroga, Bianca Arruda Manchester de Queiroga

**Affiliations:** 1 Programa de Pós-Graduação em Saúde da Comunicação Humana, Universidade Federal de Pernambuco – UFPE - Recife (PE), Brasil.; 2 Departamento de Fonoaudiologia, Universidade Federal de Pernambuco – UFPE - Recife (PE), Brasil.; 3 Curso de Fonoaudiologia, Centro Universitário de João Pessoa – UNIPÊ - João Pessoa (PB), Brasil.

**Keywords:** Linguagem Infantil, Desenvolvimento Infantil, Programa de Rastreamento, Estudo de Validação, Psicometria, Fonoaudiologia

## Abstract

**Objetivo:**

Determinar evidências de validade de critério concorrente e preditiva do Instrumento de Rastreio da Comunicação de crianças de 0 a 36 meses (IRC-36).

**Método:**

Participaram da pesquisa 78 pais/responsáveis de crianças que frequentam o serviço de puericultura das Unidades de Saúde da Família, além de 33 crianças com idades entre 0 e 36 meses, convidadas para segunda etapa do estudo. Na primeira etapa do estudo, 13 profissionais de saúde foram treinados para realizar a aplicação do IRC-36 nos pais/responsáveis das crianças. No segundo momento, os pais responderam a uma nova aplicação do IRC-36 e as crianças foram avaliadas com o Denver II.

**Resultados:**

O IRC-36 apresentou correlação com o Denver II em mais da metade dos casos, confirmando a validade de critério concorrente do instrumento. Os resultados do IRC-36 da primeira etapa quando correlacionados com o Denver II, não apresentaram valores significativos. O valor de ponto de corte do instrumento foi 12, sendo este o valor de referência entre crianças em risco e sem risco para alteração da comunicação. O instrumento apresentou valor de acurácia dentro dos níveis preconizados e alta sensibilidade. A ocorrência de risco para alteração da comunicação apresentou-se maior na segunda aplicação do IRC-36.

**Conclusão:**

O estudo apresentou evidências de validade de critério concorrente, indicando que o instrumento possui evidências de medidas de acurácia e de validade para o rastreio da comunicação de crianças de 0 a 36 meses sendo capaz de identificar risco para as alterações da comunicação.

## INTRODUÇÃO

O acompanhamento do crescimento e desenvolvimento infantil é um dos principais eixos preconizados pelo Ministério da Saúde na estratégia de atenção à saúde da criança^(1)^. No âmbito da vigilância do desenvolvimento, o monitoramento do desenvolvimento da audição e da linguagem são marcos importantes para o acompanhamento do desenvolvimento infantil, juntamente com o desenvolvimento motor^(2)^.

Os instrumentos de rastreio são importantes ferramentas para identificar atrasos no desenvolvimento, entretanto, pela ausência de instrumentos precisos e viáveis, estes não são regularmente utilizados no monitoramento do desenvolvimento da linguagem das crianças^(3)^. No Brasil, existem alguns instrumentos de avaliação da linguagem infantil, entretanto, instrumentos de rastreio ainda são escassos^(4)^.

Os testes de rastreio permitem identificar pessoas que têm um acometimento ou doença, mas que ainda não apresentam sintomas, facilitando o diagnóstico, o tratamento e reduzindo os efeitos da condição em longo prazo^(5)^. No caso de crianças, a ausência de identificação precoce, e o consequente diagnóstico tardio, dificultam o acesso ao tratamento e trazem maiores prejuízos para a qualidade de vida e desenvolvimento posterior^(6)^.

Os testes de rastreio apresentam como grande vantagem o fato de serem rápidos, de fácil aplicação, além de geralmente terem um baixo custo, proporcionando risco reduzido ao indivíduo submetido e garantindo resultado com boa sensibilidade^(7)^. Estes testes devem fazer parte da prática clínica dos profissionais de saúde^(8)^, contudo, é essencial que sejam elaborados levando em consideração medidas psicométricas, como validade e acurácia^(9,10)^.

O Instrumento de Rastreio da Comunicação de Crianças de 0 a 36 meses (IRC-36) foi um instrumento desenvolvido com o objetivo de rastrear a comunicação de crianças nos três primeiros anos de vida. É um instrumento simples, baseado do relato parental, que se divide em nove categorias distribuídas e organizadas por faixa etária, contendo perguntas específicas para cada fase do desenvolvimento da criança de 0 a 36 meses. Ao final de sua aplicação permite ao profissional que o aplicou, identificar como se apresenta a comunicação naquela etapa, classificando o desempenho da criança como em risco, sob atenção ou dentro do esperado^(11)^.

Para que um instrumento tenha seu uso recomendado, orienta-se que suas propriedades psicométricas sejam verificadas^(12)^. O IRC-36 passou pelas etapas de elaboração e validação de conteúdo^(11)^. O presente estudo visa verificar outros aspectos de validade. Nas etapas iniciais^(11)^, o IRC-36 apresentou alto índice de concordância entre os juízes, confirmando a sua validade de conteúdo.

A validade é uma das medidas psicométricas importantes e está dividida em três tipos: validade de conteúdo, validade de critério e validade de construto^(8)^. A validade de critério é verificada quando o resultado do teste em validação pode ser comparado a outro teste considerado ‘padrão-ouro’^(8)^ e está dividida em dois subtipos: preditiva e concorrente^(10)^. A validade de critério preditiva está relacionada com o resultado do teste no que se refere a condição do avaliado no futuro, ou seja, se o instrumento é capaz de prever desempenhos futuros e na validade de critério concorrente o resultado do teste em validação é comparado ao resultado de outro teste aplicado paralelamente^(10).^

Outra medida psicométrica importante é a acurácia. Esta medida está relacionada aos valores de sensibilidade e especificidade do teste em validação quando comparadas a outro teste considerado padrão de referência^(10).^

Reconhecendo a importância das medidas psicométricas na validação de instrumento^(9)^ e as etapas de validação^(10),^ o presente estudo se propôs a dar continuidade a validação do IRC-36, objetivando determinar evidências de validade de critério e de acurácia (sensibilidade e especificidade) do Instrumento de Rastreio da Comunicação de crianças de 0 a 36 meses (IRC-36).

## MÉTODO

A pesquisa foi aprovada pelo Comitê de Ética do Centro de Ciências da Saúde da Universidade Federal de Pernambuco com o número de CAAE: 26176319.6.0000.5208, de acordo com a resolução do Conselho Nacional de Saúde. Todos os indivíduos participantes, após serem esclarecidos sobre a pesquisa, assinaram o Termo de Consentimento Livre e Esclarecido. Trata-se de um estudo exploratório de validação de instrumento com abordagem quantitativa.

O estudo foi realizado em duas etapas. A primeira etapa foi realizada em quatro Unidades de Saúde da Família (USF), selecionadas por conveniência. A participação das unidades foi autorizada pela secretaria de saúde do município. Primeiramente houve a apresentação da pesquisa na reunião de equipe da ESF e os profissionais que desejaram participar do trabalho passaram posteriormente por treinamento, sendo estes os profissionais que aceitaram: dez Agentes Comunitários de Saúde (ACS) e três enfermeiras. O treinamento foi agendado na unidade em que os profissionais estavam vinculados e eles precisavam comparecer para receber o treinamento sobre a aplicação do IRC-36. O treinamento se deu por meio de leitura e explicação de cada item do IRC-36 onde os ACS e enfermeiros tiraram as dúvidas para maior segurança na aplicação.

Após o treinamento, os profissionais aplicavam o IRC-36 aos pais/responsáveis pelas crianças em sua rotina de trabalho. As enfermeiras no serviço de puericultura e os ACS na visita domiciliar.

O IRC-36 é dividido em nove categorias. Cada categoria compreende uma faixa-etária (**categoria 1**: 0 a 3 meses; **categoria 2**: 4 a 6 meses; **categoria 3**: 7 a 9 meses; **categoria 4**: 10 a 12 meses; **categoria 5**: 13 a 15 meses; **categoria 6**: 16 a 18 meses; **categoria 7**: 19 a 24 meses; **categoria 8**: 25 a 30 meses; e **categoria 9**: 31 a 36 meses).

As categorias possuem dez perguntas fechadas e as respostas possíveis para cada pergunta são: sim, não e às vezes. As respostas “sim” pontuam dois, as “às vezes” pontuam um e as “não” não pontuam. Ao final de cada pergunta é oferecido um espaço para respostas complementares todas as vezes que responderam “sim” ou “às vezes”. A pontuação total para as categorias é de 20 pontos sendo este o melhor resultado, e, o resultado final é: “**em risco”**, para as crianças que obtiveram pontuação igual ou inferior a 10, **“sob atenção”**, para as que apresentaram pontuação entre 11 e 14 pontos e **“fora de risco”** para as que apresentaram pontuação de 15 a 20 pontos.

Finalizadas as aplicações, os instrumentos eram enviados para fonoaudióloga pesquisadora para posterior análise. Foram recebidas no total 92 avaliações, entretanto, 23 instrumentos precisaram ser excluídos, pois não estavam preenchidos corretamente. Restaram então 69 instrumentos na primeira etapa.

Na segunda etapa do estudo, para verificação da validade de critério concorrente e preditiva, entrou-se em contato com os pais/responsáveis para que recebessem a reaplicação do IRC-36 e suas respectivas crianças fossem submetidas à aplicação do Teste de Triagem do Desenvolvimento Denver II.

Do total de pais/responsáveis que participaram da primeira etapa, apenas 24 retornaram para a etapa seguinte. Os motivos da perda amostral foram vários, mas os principais foram: mudança do usuário de território, não se conseguiu contato ou não compareceram no dia agendado para avaliação.

As avaliações foram realizadas no mesmo dia, uma seguida da outra e ocorreram na USF ou na residência da criança, após assinatura do TCLE pelo responsável. Para avaliação da segunda etapa, utilizou-se o critério de cegamento para a fonoaudióloga avaliadora (com relação ao resultado obtido na aplicação do primeiro IRC-36) e o teste Denver II como critério.

### Denver II

O Teste de Triagem de Desenvolvimento Denver II é um instrumento que permite avaliar crianças de zero a seis anos, quanto ao seu desenvolvimento nas áreas pessoal-social (socialização da criança), motora fina-adaptativo (coordenação olho-mão), linguagem (reconhecer, entender e usar a linguagem) e motora grossa (controle motor corporal), e, são verificados mediantes observação direta da criança.

Os itens do Denver II devem ser visualizados com a criança desempenhando determinada tarefa com possibilidade de passa (P), falha (F) ou recusa (R). Caso não seja possível visualizá-las executando a tarefa, alguns itens permitem que o responsável relate se a criança já o desempenha, se teve ou não oportunidade de desempenhá-lo. No Denver II as crianças podem apresentar desenvolvimento normal ou risco para atraso no desenvolvimento.

Para aplicação do Denver II, fez-se necessário que a fonoaudióloga avaliadora realizasse um curso de treinamento com duração de 70 horas para certificação de aplicador. A profissional recebeu treinamento teórico e prático, através de aulas online, leitura e discussão de artigos, vídeos explicativos e exercícios práticos. Também foi necessária a aquisição do “kit de teste” padronizado para evitar redução da confiabilidade dos resultados obtidos.

Após aplicação dos testes os pais recebiam os resultados e eram orientados quanto à estimulação da linguagem oral e quando necessário, a criança era encaminhada para a rede especializada.

Este estudo foi realizado em um dos municípios da Região Metropolitana de Recife. Os profissionais aplicadores foram, três enfermeiras e dez agentes comunitários de saúde na primeira etapa e a fonoaudióloga pesquisadora na segunda etapa. Participaram da pesquisa 78 pais/responsáveis de crianças que frequentam o serviço no qual esses profissionais estão inseridos, em sua grande maioria mães (99%), além de 33 crianças com idades entre 0 e 36 meses, convidadas para a segunda etapa do estudo.

Dos 78 protocolos IRC-36 aplicados, 41 (52,6%) eram de crianças do sexo feminino e 37 (47,4%) do sexo masculino. A média de tempo entre o primeiro e o segundo IRC-36 foi de cinco meses e três dias. As aplicações estão descritas no Quadro 1.

**Quadro 1:** Aplicação dos instrumentos por profissional, local de aplicação e quantitativo de instrumentos aplicados

Aplicador	Local de aplicação	Aplicação do IRC-36 (1ª etapa)	Aplicação do IRC-36 + Denver II (2ª etapa)		Total
			Reaplicação do IRC-36 + Denver II	Aplicação do IRC-36 + Denver II	
ACS	Domicílio (visita domiciliar)	57	-	-	57
Enfermeiro	USF (Puericultura)	12	-	-	12
Fonoaudióloga avaliadora	USF ou domicílio	-	24	9	33
Total		69	24	9	102

**Legenda:** ACS = Agente Comunitário de Saúde; USF = Unidade de Saúde da Família; IRC = Instrumento de Rastreio da Comunicação.

Utilizou-se como critérios de exclusão deste estudo, pais/ responsáveis que apresentem limitação intelectual, pais/ responsáveis com idade inferior a 18 anos, pais de crianças com alterações genética ou neurológica e crianças com diagnóstico de problema de audição e/ou visão.

Os dados coletados foram organizados e analisados por meio do programa estatístico SPSS, versão 21.0. Depois da verificação da não normalidade da distribuição dos dados, aplicaram-se os testes não-paramétricos Exato de Fisher e de Correlação de Spearman para avaliar as associações e as correlações. O nível de significância considerado para o cálculo foi de 5%.

Dos 69 pais que responderam ao primeiro IRC-36, 24 deles receberam a reaplicação do instrumento e o Denver II, e, nove novas crianças que entraram na amostra, os pais responderam apenas o segundo IRC-36 e o Denver II. Totalizou-se 102 IRC aplicados e 33 testes Denver II.

É importante salientar que diante do cenário de pandemia do Covid-19 vivenciado nos últimos anos a coleta teve atraso em seu início e seu ritmo sofreu influência em consequência das dificuldades enfrentadas no contexto pandêmico. Entretanto, mesmo diante de todas as dificuldades enfrentadas foi possível coletar dados suficientes para conclusão da pesquisa. Segue abaixo na Figura 1 o fluxograma da coleta de dados.

**Figura 1:** Fluxograma metodológico da coleta dos dados

Fonte: A autora (2022)

## RESULTADOS

A Tabela 1, a seguir, apresenta o cruzamento dos desempenhos das crianças no IRC-36 1ª (primeira aplicação, realizada pelos ACS e enfermeiras), no IRC-36 2ª (segunda aplicação realizada pela pesquisadora responsável) e no Teste de Denver II. Para análise, considerou-se “esperado” o desempenho de crianças **fora de risco** e “não esperado” o desempenho de crianças cujos scores no IRC indicavam estar **sob atenção** ou **em risco** para as alterações da comunicação.

**Tabela 1:** Dados referentes à classificação do desempenho das crianças no IRC e no Denver II Total

**Variável**		**RESULTADO DENVER II**						**p-valor**
		**NORMAL**		**RISCO**		**Total**		
		**n**	**%**	**N**	**%**	**N**	**%**	
IRC-36 FINAL 1	Fora de Risco	7	58,3%	7	58,3%	14	58,3%	0,435
	Sob Atenção	4	33,3%	2	16,7%	6	25,0%	
	Em Risco	1	8,3%	3	25,0%	4	16,7%	
	Total	12	50,0%	12	50,0%	24	100,0%	
IRC-36 FINAL 2	Fora de Risco	**15**	**83,3%**	4	26,7%	19	57,6%	0,002
	Sob Atenção	3	16,7%	**6**	**40,0%**	9	27,3%	
	Em Risco	0	0,0%	5	33,3%	5	15,2%	
	Total	18	54,5%	15	45,5%	33	100,0%	
IRC-36 1ª	Esperado para idade	11	91,7%	9	75%	20	83,3%	0,295
	Não esperado	1	8,3%	3	25%	4	16,7%	
	Total	12	100,0%	12	100,0%	24	100,0%	
IRC-36 2ª	Esperado para idade	**15**	**83,3%**	4	26,7%	19	57,6%	0,0001
	Não esperado	3	16,7%	**11**	**73,3%**	14	42,4	
	Total	18	54,5%	15	45,5%	33	100,0%	

Teste Exato de Fisher; significância p<0,05*

**Legenda:** IRC-36 FINAL 1 = resultado pelas três estratificações do IRC-36 na primeira aplicação; IRC-36 FINAL 2 = resultado pelas três estratificações do IRC-36 na segunda aplicação; IRC-36 1ª = IRC-36 na primeira aplicação; IRC-36 2ª = IRC-36 na segunda aplicação.

Como é possível observar, o desempenho das crianças na segunda aplicação do IRC-36 se associou à classificação do Denver II em 83,3%, o que significa dizer que as classificadas fora de risco pelo IRC-36 também estavam dentro da faixa considerada normal para o Denver II, confirmando a validade de critério concorrente do instrumento.

Além disso, também se observou associação significativa (73,3%) entre a frequência de casos com desempenho “não esperado” (incluídos aqui os desempenhos em risco e sob atenção) para a idade no IRC-36 e os casos de risco no Denver II. Estes resultados demonstram boa sensibilidade do IRC-36 e reforçam sua validade de critério concorrente.

Contudo, como é possível visualizar na Tabela 1, não foram observadas associações significativas entre os escores obtidos na primeira aplicação do IRC-36 e o teste Denver.

A fim de aprofundar essa questão e se seriam obtidos resultados diferentes com a manutenção dos scores originais do IRC-36, sem considerar a classificação de crianças **sob atenção** e **em risco** como “não esperado”, foi realizada uma análise de Correlação de Spearman. Esta análise pode ser visualizada na Tabela 2, a seguir.

**Tabela 2:** Matriz de correlação (Spearman´s rho) entre o desempenho no primeiro e segundo IRC-36 com o Denver II e com o domínio linguagem do Denver II

Testes	Denver II	Denver II – Linguagem
1º IRC-36	0,752	0,715
2º IRC-36	0,606**	0,621**

** A correlação é significativa no nível p<0,01

Como é possível observar mais uma vez, encontrou-se uma forte correlação entre a segunda aplicação do IRC-36 e o Denver II (rho = 0,606), confirmando a validade concorrente de que o instrumento avalia aspectos de linguagem e pode ser comparado com uma medida de referência o Denver II. As aplicações também foram correlacionadas com a área específica de linguagem do Denver II e a apresentaram forte correlação (rho = 0,621; p<0,01). Também de modo semelhante ao observado anteriormente, a primeira aplicação do IRC-36 não apresentou correlação significativa com o Denver II.

Os resultados do IRC-36 1ª quando correlacionados com o Denver II na classificação padrão e no resultado “esperado” e “não esperado” não apresentaram valores significativos, não sendo possível, com esses resultados, confirmar a capacidade preditiva do instrumento.

Considerando que o teste de Denver possui os seguintes domínios: pessoal-social, motor fino adaptativo, linguagem e motor grosso, com o objetivo de explorar mais a fundo as relações entre o IRC-36 e o domínio “linguagem” do Denver, foi realizada uma nova análise de associação por meio do Teste Exato de Fisher e pode ser visualizada na Tabela 3.

**Tabela 3:** Dados referentes à classificação do desempenho das crianças no IRC e no Denver II linguagem

**Variável**		**RESULTADO DENVER LINGUAGEM**						p-valor
		**NORMAL**		**RISCO**		**Total**		
		**N**	**%**	**N**	**%**	**N**	**%**	
IRC-36 FINAL 1	Fora de Risco	**11**	**61,1%**	3	50,0%	14	58,3%	0,853
	Sob Atenção	4	22,2%	2	33,3%	6	25,0%	
	Em Risco	3	16,7%	1	16,7%	4	16,7%	
	Total	18	75,0%	6	25,0%	24	100,0%	
IRC-36 FINAL 2	Fora de Risco	**15**	**83,3%**	4	26,7%	19	57,6%	0,002
	Sob Atenção	3	16,7%	**6**	**40,0%**	9	27,3%	
	Em Risco	0	0,0%	5	33,3%	5	15,2%	
	Total	18	54,5%	15	45,5%	33	100,0%	
IRC-36 1ª	Esperado para idade	15	83,3%	5	83,3%	20	63,6%	0,749
	Não esperado	3	16,7%	1	16,7%	4	36,4%	
	Total	18	75%	6	25%	24	100,0%	
IRC-36 2ª	Esperado para idade	**18**	**94,7%**	1	5,3%	19	57,6%	0,002
	Não esperado	6	42,9%	**8**	**57,1%**	14	42,4%	
	Total	24	72,7%	9	27,3%	33	100,0%	

Teste Exato de Fisher; significância p<0,05*

**Legenda:** IRC-36 FINAL 1 = resultado pelas três estratificações do IRC-36 na primeira aplicação; IRC-36 FINAL 2 = resultado pelas três estratificações do IRC-36 na segunda aplicação; IRC-36 1ª = IRC-36 na primeira aplicação; IRC-36 2ª = IRC-36 na segunda aplicação

Na relação entre a classificação do IRC-36 e o Denver II referente à Linguagem, observou-se um resultado semelhante, sendo que a associação foi ainda maior (94,7%) quando observados os casos com desenvolvimento “esperado” e “não esperado” em relação à normalidade e ao risco. De modo semelhante ao que foi observado anteriormente, a primeira aplicação do IRC-36 novamente não apresentou correlação significativa com o Denver II no domínio linguagem.

A verificação da confirmação dos pontos de corte do instrumento para permitir discriminar os indivíduos com os desfechos propostos também foi verificada, considerando-se “esperado” quando os resultados foram **fora de risco** e “não esperado” quando foram **sob atenção** ou **em risco**.

A Figura 2 aponta a curva ROC com uma área sob a curva de 0,796 (IC95%: 0,634 - 0,959).

**Figura 2:** Curva ROC IRC 2 a partir da classificação Denver II – normal e risco

Fonte: A autora (2022)

Considerou-se para o melhor valor de corte o ponto onde existe o menor erro possível e melhor medida de sensibilidade, sendo o melhor valor 11,5. Contudo, como no IRC-36 as pontuações são mediante números inteiros, o valor do ponto de corte será considerado 12. Levando em consideração que o IRC-36 é um instrumento de rastreio, o instrumento indicará presença de risco de alteração da comunicação quando o valor for menor que 12 e ausência de risco de alteração quando o valor for igual ou maior que 12.

A acurácia do teste é representada pela área da curva e foi de 79,6% (Tabela 4) e está dentro dos níveis preconizados, confirmando que o instrumento é capaz de prever crianças com e sem risco de alteração da comunicação. O valor de sensibilidade do instrumento foi de 94,4%, sendo este um alto valor a ser considerado em instrumentos de rastreio, pois verifica a capacidade do instrumento em detectar pessoas que provavelmente têm a condição investigada.

**Tabela 4:** Área sob a Curva e coordenadas da curva ROC IRC-36 2ª

**Área**	**Desvio Padrão** a	**Nível de significância P (Área=0.5)** b	**Intervalo de confiança assintótico 95%**		**Critério de associação**	**Sensibilidade (%)**	**Especificidade (%)**
			**Limite Inferior**	**Limite superior**			
0,796	0,083	<0,004	0,634	0,959	11,50	94,4	60,0

a DeLong et al.^(13)^

b Binomial exact

Na primeira aplicação do IRC-36 obteve-se um grande quantitativo de crianças “sob atenção” e “em risco” para alteração da comunicação, sendo esta ocorrência de 13% na primeira aplicação e 15,2% na segunda aplicação. Quando considerado o resultado **não esperado** que soma os resultados “em risco” e “sob atenção”, esses valores aumentaram sendo 36,2% e 42,5%, respectivamente.

Com relação às áreas do Denver II, o domínio linguagem foi o que apresentou o maior número de crianças em risco para atraso (27,3%), seguido do domínio pessoal-social (24,2%). Quando considerado o resultado geral do Denver II, ou seja, levando em consideração todos os domínios do instrumento, o quantitativo de crianças em risco para alteração do desenvolvimento foi de 45,5%.

O desempenho ao longo do tempo das 24 crianças que realizaram as duas aplicações do IRC-36 está exposto na Figura 3.

**Figura 3:** Desempenho das 24 crianças no IRC-36 primeira aplicação e IRC-36 segunda aplicação ao longo do tempo

Fonte: A autora (2022)

Das 24 crianças avaliadas em momentos distintos, no primeiro IRC-36 quatro estavam “em risco” e seis “sob atenção”. No segundo IRC-36 três ficaram “em risco” e sete “sob atenção”. Duas crianças que estavam em risco no primeiro IRC, permaneceram no segundo momento, a que estava “sob atenção” desceu para o risco, uma continuou no sob atenção. As médias nos dois momentos foram de 14,95 e 14,37, sendo a média do segundo IRC-36 menor.

Entre as 33 crianças rastreadas com o IRC-36 e o Denver II, duas foram encaminhadas ao serviço especializado, pois apresentaram risco nas duas avaliações, atraso de linguagem considerável, além de sinais de Transtorno do Espectro do Autismo como: não atender quando chamadas pelo nome, dificuldade de interação, estereotipias vocais e ausência de contato visual.

## DISCUSSÃO

Os resultados da presente pesquisa apontam correlação positiva entre o IRC-36 e o Denver II, confirmando a validade de critério concorrente. Considerando que o Denver II é um instrumento conceituado internacionalmente, que vem sendo amplamente utilizado na prática clínica e em pesquisas brasileiras^(14,15)^, além de vir passando por etapas de validação com resultados positivos^(16,17)^, sua correlação com o IRC-36 aponta o quão válido é o novo instrumento e quão útil ele pode ser para verificar os aspectos da comunicação, tendo em vista a escassez de instrumentos de triagem da linguagem infantil no Brasil^(4)^.

A verificação da validade de critério preditiva não apresentou valores significativos, ou seja, pelo menos na presente investigação o instrumento não foi capaz de predizer o resultado futuro do desempenho da comunicação da criança. Contudo, é importante destacar que alguns aspectos podem ter interferido neste resultado, dentre eles destacam-se o tempo de aplicação entre a primeira e segunda triagem, a aplicação nas duas etapas ter sido realizada por aplicadores diferentes e a pesquisa ter sido realizada durante um período crítico da pandemia da Covid-19 que pode ter trazido modificações no desenvolvimento infantil. Estudos já mostram que o contexto de isolamento social provocado pela pandemia do COVID-19 pode gerar fatores de risco para o crescimento e desenvolvimento da criança^(18)^.

Outra questão relativa à aplicação que também deve ser levada em consideração é que o treinamento ofertado aos aplicadores pode não ter sido suficiente para capacitá-los, comprometendo assim o resultado final dos testes quando comparados. Isto pode ter ocorrido devido à metodologia aplicada no treinamento, sendo esta apenas expositiva, quando sugere-se que no processo de educação permanente se priorize o emprego de metodologias ativas, nas quais os profissionais podem vivenciar a prática, facilitando a aquisição do conhecimento^(19)^. Por outro lado, vale ressaltar que, devido à rotina do serviço e o momento de pandemia vivenciado na etapa de coleta de dados, além do tempo que é um desafio na atenção primária^(20)^ não foi possível realizar outro modelo de formação.

O valor de sensibilidade do IRC-36 (no segundo momento) quando relacionado com o Denver II no domínio linguagem, mostrou que entre os casos de risco de alteração confirmados pelo critério (Denver II) o IRC-36 foi capaz de identificar corretamente quem tem risco de alteração em 94,4% das vezes. Um estudo holandês que realizou a verificação da validade concorrente e preditiva de um teste de triagem da linguagem infantil, obteve bons resultados de validade concorrente (sensibilidade de 0,79 e especificidade de 0,86) e mostrou uma alta capacidade preditiva do instrumento (sensibilidade de 0,82 e uma especificidade de 0,74) indicando o quão importante esses instrumentos podem ser na observação da linguagem infantil, inclusive para prever resultados futuros, além de fornecerem suporte aos profissionais que atuam na saúde infantil^(21)^. Portanto, o IRC-36 pode ser utilizado com a finalidade de fornecer informações de como se encontra a comunicação da criança, além de favorecer o trabalho dos profissionais que acompanham o desenvolvimento infantil.

A curva ROC evidenciou o ponto de corte entre crianças com risco de alteração e crianças sem risco, apresentando um valor de acurácia de 79,6% estando dentro dos níveis preconizados. Quanto mais distante a curva estiver da diagonal principal, aproximando-se do canto superior esquerdo, melhor será o desempenho do teste para discriminar pessoas com e sem o desfecho investigado^(22)^.

O ponto de corte do instrumento foi verificado através da curva ROC e identificou-se o valor que delimita indivíduos com e sem risco para alteração na comunicação, sendo este de 12 (score/pontuação). Este valor faz referência ao valor da estratificação primária do IRC-36^(11)^, que define crianças “em risco” para alteração da comunicação as que pontuarem até 10 e “sob atenção” as que obtiverem pontuação entre 11 e 14. Entretanto, devido aos resultados obtidos na curva ROC que delimitaram o valor entre crianças em risco e fora de risco, a estratificação do “sob atenção” assumirá os valores de 13 e 14 para indicar crianças que precisam ser monitoradas no desenvolvimento da comunicação. Os valores de ponto de corte poderão ser melhor verificados seguindo a classificação original quando houver ampliação do N da amostra.

Tendo em vista que o IRC-36 tem como constructo principal a comunicação sua aplicação pode apresentar-se mais breve que o Denver II que por sua vez abrange quatro domínios do desenvolvimento^(23)^.

Além disso, o IRC-36 é um teste de rastreio de baixo custo em que se necessita apenas do formulário para ser preenchido e de uma orientação prévia^(11)^, atendendo as recomendações do que é preconizado para instrumentos de rastreio: rápido, de fácil aplicação e de baixo custo^(7)^, o lhe que difere do Denver II que preconiza uma formação específica para sua aplicação, além da aquisição de um “kit de teste” com os itens específicos para avaliação^(23)^.

A aplicação do instrumento em validação pode ser realizada por profissionais de saúde e educação que estão envolvidos com o acompanhamento do desenvolvimento infantil a fim de auxiliá-los no acompanhamento do desenvolvimento da comunicação da criança. Neste sentido é importante destacar, que o atraso de linguagem pode apresentar-se com alta prevalência nos três primeiros anos de vida^(24)^.

A ocorrência de risco para alteração de comunicação foi de 13% e 15,2%, nos dois momentos respectivamente. Tais resultados evidenciam que os aspectos da comunicação infantil devem ser acompanhados, tendo em vista que as alterações atuais podem gerar grandes repercussões futuras e que as alterações de linguagem quando não identificadas no momento oportuno podem influenciar o desenvolvimento infantil em todas as áreas^(25)^.

O número de crianças com risco para alteração mostrou-se alto tanto no IRC-36 quanto no Denver II. Em estudo com crianças pré-escolares avaliadas com o Denver II verificou-se 24,8% de risco de atraso no desenvolvimento^(26),^ sendo que na presente pesquisa esse valor praticamente dobrou (45,5%) corroborando com estudo realizado com usuários da Atenção Primária à Saúde com idade entre sete e 18 meses^(27)^, onde o valor de crianças em risco foi de 47,37%. Este alto percentual de risco de atraso no desenvolvimento pode estar relacionado à realidade da criança acompanhada na atenção básica no contexto estudado na presente investigação, que pode receber pouco estímulo devido as muitas atribuições que a família possui, além de terem menos oportunidade de estimulação à medida que a idade vai aumentando^(27)^. Somando-se a isso destaca-se, como mencionado anteriormente, que a pesquisa foi realizada em um período crítico da pandemia em que as crianças tiveram suas experiências sociais limitadas pelo isolamento social, fato que pode ter contribuído para o aumento na prevalência de crianças em risco^(18)^.

Outros estudos também verificaram a área de linguagem como a área que mais apresenta risco de atraso no desenvolvimento^(27,28)^ por isto, a importância de monitorar o desenvolvimento linguístico e orientar os cuidadores, tendo em vista que a aquisição de linguagem está relacionada aos estímulos que a criança recebe no meio em que vive^(29)^.

A utilização do IRC-36 pode ser útil para implementar estratégias de estimulação da comunicação infantil quando esses níveis de desenvolvimento estiverem no “sob atenção” ou “em risco”. É fundamental fornecer estratégias de intervenção para a criança e suas famílias para assim prevenir atraso no desenvolvimento, pois a estimulação familiar inadequada é um fator de risco para atraso de linguagem^(30)^.

O IRC-36 é um instrumento útil, que vem passando pelas etapas de validação orientadas, obtendo valores positivos que evidenciam o quão válido o instrumento pode ser. No presente estudo, foi possível observar outros parâmetros de validade em que o instrumento passou com resultados satisfatórios.

O instrumento irá contribuir para a verificação da comunicação infantil na atenção primária à saúde e possivelmente em outros contextos, como nas creches infantis. É um teste simples, de fácil aplicação e que tem uma importante aplicabilidade para o desenvolvimento infantil, tendo em vista que a linguagem é uma habilidade que influencia outras áreas do desenvolvimento^(31)^.

## CONCLUSÃO

O estudo mostrou que houve uma forte correlação entre o IRC-36 e o Denver II, confirmando a validade de critério concorrente do instrumento e indicando que o instrumento em validação pode ser utilizado para o rastreio de comunicação de crianças de 0 a 36 meses. O instrumento também apresentou medidas de acurácia e de sensibilidade satisfatórias, apontando que é capaz de identificar risco para as alterações da comunicação. Observou-se a ocorrência de crianças “em risco e sob atenção” para as alterações da comunicação, sendo estes valores expressivos e apontando o quanto é importante acompanhar o desenvolvimento infantil. Os valores da validade de critério preditiva não foram significativos, contudo alguns aspectos metodológicos e circunstanciais podem ter influenciado no resultado e este aspecto necessita ser melhor investigado em estudos posteriores.

Levando em consideração o momento de pandemia do Covid-19 no qual a presente pesquisa foi realizada, além da diferença de tempo entre a primeira e a segunda aplicação e sendo estas realizadas por profissionais diferentes, sugere-se, ainda, para os trabalhos futuros uma nova aplicação do instrumento após esse período pandêmico para confirmação dos resultados obtidos anteriormente, redução e padronização do tempo de aplicação entre as etapas do estudo, ampliação da amostra e diversificação do seu contexto social e geográfico da população testada, tendo em vista que a pesquisa foi realizada apenas na Atenção Primária à Saúde com um público de um nível socioeconômico baixo. Outra relevante sugestão é verificar junto aos aplicadores as dificuldades encontradas na realização da triagem, além do tempo médio de aplicação.
